# Dataset of vector mosquito images

**DOI:** 10.1016/j.dib.2022.108573

**Published:** 2022-09-07

**Authors:** Reshma Pise, Kailas Patil, Meena Laad, Neeraj Pise

**Affiliations:** a Research scholar, Vishwakarma University, Pune, India; b Professor, Vishwakarma University, Pune, India; c Professor, Symbiosis Institute of Technology, Pune, India; d Graduate student, Computer Science , Rutgers University, New Brunswick NJ, United States

**Keywords:** Computer vision, Deep learning, Mosquito classification, Vector control

## Abstract

Mosquitoes pose substantial threat to public health resulting in million number of deaths wordlwide every year. They act as the vectors responsible for diseases such as Dengue, Yellow fever,Chikungunya, Zika etc. The harmful mosquito species are contained in the genera Aedes, Anopheles and Culex. Automated species identification of vectors is essential to implement targeted vector control strategies. The objective of the proposed paper is to construct a novel dataset of images of dangerous mosquito species. We have prepared a dataset of images of adult mosquitoes belonging to three species: Aedes Aegypti, Anopheles stephensi and Culex quinquefasciatus stored in two folders. The first folder comprises of total 2640 augmented images of mosquitoes belonging to the three species. The second folder contains original images of the the three species. The dataset is valuable for training machine and deep learning models for automatic species classification.

**Specifications Table**

**Table:** 

Subject:	Computer Vision and Pattern Recognition, Machine Learning, Entomology and insect science.
Specific subject area:	Morphological classification of mosquito species.
Type of data:	Images of Mosquitoes
How data points were acquired:	The images were captured with a 48 Mpx One Plus mobile phone camera in the day light condition.
Data format:	Raw images in JPEG file format.
Description of data collection:	Photographs of fresh mosquito specimens were shot at day light using high resolution mobile phone rear camera.
Data source location:	All photos were captured at Ross life Lab located in the city of Pune, India ROSS LIFE SCIENCE PVT. LTD Plot No.96, Sector No.10, PCNTDA, Bonsai, Pune – 411026 Maharashtra, India Latitude and longitude: 18.6466° N, 73.8306° E
Data accessibility:	The dataset of images is available online Mendeley website. Repository name: Dataset of Vector Mosquito Images Data identification number (doi): 10.17632/88s6fvgg2p.4 Direct URL to data: https://data.mendeley.com/datasets/88s6fvgg2p/4

## Value of the Data

• The dataset provides images of three mosquito species: Aedes Aegypti, Anopheles stephensi and Culex quinquefasciatus.
• The dataset can be used to train mosquito species classification and prediction models. The dataset can potentially benefit the society in controlling mosquito borne diseases.
• The dataset can be used to train automated species classification models which is a vital contribution for vector control.
• Automated genera and species identification can be efficient as compared to the laborious and time consuming task of manual species identification carried out by entemologists.

## 1 Data Description

Mosquitoes of genera Aedes, Anopheles and Culex are vectors responsible for spreading diseases such as Dengue, Yellow fever, Chikungunya, Zika etc. [1]. Mosquito vector surveillance is carried out by local government to monitor the mosquito population and the species predominant in a geographic area to implement effective mosquito vector control plans [2]. Automated species classification can be an important contribution to target harmful species. Image processing techinques with machine learning algorithms can be used to train machine learning models to classify and predict the genera or species. Availability of a quality data set is a prerequisite to train such deep learning models [3,^4^,5].

There are datasets which include geographical density and distribution record of vector mosquito species [6,7]. There are images datasets available which contain images of female mosquitoes belonging to i) Aedes genera (Aedes aegypti and Aedes albopictus species), ii) Aedes and Culex genera (Aedes aegypti and Aedes albopictus and Culex quinquefasciatus species and  iii) Aedes, Anopheles and Culex species. [8,^9^,10].

The images in these datasets were acquired using microscope and a digital camera. In our work we have included the three important harmful vector species i.e., Aedes aegypti, Anopheles stephensi and Culex quinquefasciatus of both sexes. Also, the images are captured with mobile phone camera.

The proposed dataset folder comprises of two folders. The folder named “Mosquito Images Original” contains original images of the three species stored under three sub folders that are created for each of the three species: Aedes Aegypti, Anopheles Stephensi and Culex Quinquefasciatus. The folder named “Mosquito Images Augmented” consists of total 2640 augmented images of the three species stored under the 3 corresponding subfolders. The pictures were captured with One Plus mobile 48 Mpx camera and were saved in JPEG file format. The original pictures are RGB images with dimension 3000 x 4000 pixels and 72 dpi. The augmented images are of resolution 256 × 256 pixels with 96 dpi. Table 1presents the number of images and a sample picture of mosquito belonging to each of the three species..

**Table 1:** Dataset description

SubFolder	Number of original Images	Number of Augmented Images	Sample Image
Aedes Aegypti	15	900	
Anopheles Stephensi	9	540	
Culex Quinquefasciatus	20	1200	

## 2 Experimental Design, Materials and Methods respective

### 2.1 Experimental Design

**Fig. 1:**
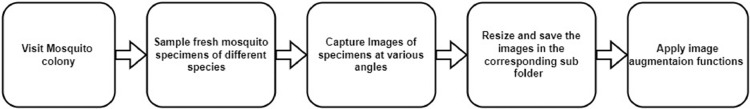
Data acquisition process

The images were collected during April 2022, at the mosquito colony maintained by Ross Life lab, Pune city of Maharashtra, India. Fig. 1 shows the steps involved in dataset construction process. The fresh adult mosquito specimens were imaged using a handheld smartphone camera. The species included were Aedes Aegypti, Anopheles Stephensi and Culex Quinquefasciatus. The species of the specimen were confirmed by an entomologist from the Ross Life.

### 2.2 Materials or Specification of Image Acquisition System

The imaging system consisted of a 48 Mpx One Plus mobile RGB camera. (Table. 2)

**Table 2:** Specification of image acquisition system

Sr. No.	Particulars	Details
1	Camera	a) Make and Model: Sony IMX586 and
		b) Sensor: 48 MP
		b) Focus Adjustment: automatic
		c) Aperture: f / 1.7

2	Resolution of augmented images	256 × 256 pixels

3	Image Format	JPEG

4	Original Image Resolution Range	3000 × 4000

### 2.3 Method

Adult mosquitoes of both sexes belonging to the three species were sampled and photographed. There were 7 Aedes Aegypti, 5 Anopheles and 10 Culex specimens. The genera and species of the mosquitoes were confirmed by an entomologist. The live mosquito specimens were frozen and placed over a plain white surface. Specimens were oriented slightly and images were taken at various angles to capture morphological features specific to each species. This was easy to achieve with fresh specimens.

The images were stored with the file name format as: genus_species_imagenumber.jpg in the corresponding species subfolder under “Mosquito Images Original” folder. The data augmentation procedure was used to increase the size of the dataset as huge dataset is a requirement for machine learning projects. It is a is a well-known technique in image classification problems to make the model generalize better. The original images were resized to 256 × 256 pixels which is a standard input image resolution for deep neural networks. Image augmentation functions from python library “imgaug” were used to augment all the original images. Augmentation functions such as random rotations, scale, shears, perspective transformation, flips, gaussian blur, noise and colour space transformations were applied with different parameters. For example rotation augmentation was performed by rotating the image by 10°, between 10° - 360°. Table 3 presents the augmentation functions, parameters and parameter values that we used for each function. These parameter values can be changed to get an altered set of new images from original images dataset.

**Table 3:** Image Augmentation details

Augmentation function	Parameter Values
Rotation	Degree of rotation: 10°, between 10° - 360°
Scaling	Scaling Factor: 1.2 and 0.8
Shear	Between 16 °- 16 °
Perspective	Scale: 0.15
Gaussian Blur	Sigma value =1
Flip	Horizontal flip for all images
Gaussian Noise	Scale: (0, 0.05 * 255)
GammaContrast	Pixel Values in range: 0.5 and 1.44
Linear Contrast	Pixel Value: 0.62

The resulting image files are named genus_species_ imagenumber.jpg and stored in the respective subfolders in the “Mosquito Images Augmented ” folder. The number of images in each category afer augmentation is specified in Table 1

## Ethics Statement

This data is available in the public domain, and no funding is received for the present effort. There is no conflict of interest.

## CRediT Author Statement

**Reshma Pise:** Conceptualization, Writing the original article, Publication of the dataset; **Kailas Patil:** Data Validation, Supervision, Project administration; **Meena Laad:** Editing, Formal analysis; **Neeraj Pise:** Image capture & Augmentation.

## Declaration of Competing Interest

The authors declare that they have no known competing financial interests or personal relationships that could have appeared to influence the work reported in this paper

## Data Availability

Dataset of Vector Mosquito Imageshttps://data.mendeley.com/datasets/88s6fvgg2p/1 (Original data) (Mendeley Data)
